# Are the first 1,000 days of life a neglected vital period to prevent the impact on maternal and infant morbimortality of infectious diseases in Latin America? Proceedings of a workshop of experts from the Latin American Pediatric Infectious Diseases Society, SLIPE

**DOI:** 10.3389/fped.2023.1297177

**Published:** 2023-11-30

**Authors:** Roberto Debbag, Jaime R. Torres, Luiza H. Falleiros-Arlant, Maria L. Avila-Aguero, Jose Brea-del Castillo, Angela Gentile, Xavier Saez-Llorens, Abiel Mascarenas, Flor M. Munoz, Juan P. Torres, Liliana Vazquez, Marco A. Safadi, Carlos Espinal, Rolando Ulloa-Gutierrez, Monica Pujadas, Pio Lopez, Eduardo López-Medina, Octavio Ramilo

**Affiliations:** ^1^ President of Sociedad Latinoamericana de Infectología Pediátrica, SLIPE, Buenos Aires, Argentina; ^2^ Infectious Diseases Section, Tropical Medicine Institute, Universidad Central De Venezuela, Caracas, Venezuela; ^3^ Department of Children’s Health, Faculdade De Medicina, Universidade Metropolitana De Santos, Santos, Brazil; ^4^ Infectious Diseases Service, Hospital Nacional De Niños “Dr. Carlos Sáenz Herrera”, Caja Costarricense De Seguro Social (CCSS), San José, Costa Rica; ^5^ Affiliated Researcher Center for Infectious Disease Modeling and Analysis (CIDMA) at Yale University, New Haven, CT, United States; ^6^ Associated Researcher, Investigador Asociado Hospital Dr. Hugo Mendoza, Santo Domingo, Republic Dominicana; ^7^ Epidemiology Department, Hospital de Niños “Ricardo Gutiérrez”, Buenos Aires University, Buenos Aires, Argentina; ^8^ Head of Infectious Diseases and Director of Clinical Research, Hospital del Niño “Dr. José Renán Esquivel”, Panama City, Panama; ^9^ Department of Pediatric Infectious Diseases, Hospital Universitario “José E. Gonzalez”, Universidad Autónoma De Nuevo León, Nuevo Leon, México; ^10^ Department of Pediatrics, Baylor College of Medicine, Houston, TX, United States; ^11^ Department of Pediatrics and Children Surgery, Universidad de Chile, Santiago, Chile; ^12^ Pediatric Infectious Diseases, Clinica y Maternidad Suizo Argentina, Sanatorio Finochietto, Buenos Aires, Argentina; ^13^ Department of Pediatrics, Faculda de de Ciências Médicas da Santa Casa de São Paulo, Sao Paulo, Brazil; ^14^ Global Health Consortium, Robert Stempel College of Public Health & Social Work, Florida International University, Miami, FL, United States; ^15^ Department of Epidemiology and Pediatric Infectious Diseases, Centro Hospitalario Pereira Rossell, Faculty of Medicine, Universidad de la República, Montevideo, Uruguay; ^16^ Department of Pediatrics, Hospital Universitario del Valle, Cali, Colombia; ^17^ Centro de Estudios en Infectología Pediátrica CEIP, Department of Pediatrics, Universidad del Valle, Clinica Imbanaco Grupo Quironsalud, Cali, Colombia; ^18^ Department of Infectious Diseases, St. Jude Children’s Research Hospital, Memphis, TN, United States

**Keywords:** first 1,000 days of life, pregnancy immunization, infant immunization, pediatric, social determinants of health

## Abstract

While the first 1,000 days of life are a critical period in child's development, limited information on the main determinants affecting this period in the Latin America and the Caribbean (LAC) region is available. Therefore, the Latin American Pediatric Infectious Diseases Society (SLIPE) held an *ad hoc* workshop in May 2022 with an expert panel designed to analyze the main factors impacting the development of childhood in the region during this period and the main causes of maternal infant morbimortality. The aim was to identify priorities, generate recommendations, and advise practical actions to improve this situation. Considerations were made about the challenges involved in bridging the gap that separates the region from more developed countries regarding an optimal early childhood and maternal care. Extensive discussion was conducted to reach consensus recommendations on general strategies intended to reduce maternal and infant mortality associated with infections and immune-preventable diseases during the first 1,000 days of life in LAC.

## 1. Introduction

The first 1,000 days of life—the time spanning between the moment a woman becomes pregnant and her child's second birthday—is a unique window of opportunity where foundations for children growth, ability to learn, and health are built (1, 2). This period is considered the phase where brain begins its development, and the base of future health and neurodevelopment. It also represents a time of vulnerability. For example, nutrition of the mother during pregnancy and the one a child receives in the first two years of life, is a crucial determinant of health (1–3). In low-income countries (LIC), poverty often debilitates access to proper food, causing mortality, significant morbidities, and a substantial loss of neurodevelopmental potential (2, 3).

During the last decades, plentiful research in the fields of neuroscience, biology, and early childhood development provided powerful insights into how interaction between nutrition and environments in these 1,000 days shape future outcomes.

The first 1,000 days are characterized by rapid rates of neuronal proliferation, growth and differentiation, myelination, and generation of synapses. This is also a time of brain vulnerability to any nutrient deficit, infectious diseases, detrimental living conditions, or vaccination status among others.

Nutrition plays a fundamental role in child's development. Poor nutrition in the first 1,000 days and its sequelae can cause irreversible damage to a children's growing brain, affecting proper education, and making it harder for them and their families to rise from poverty. It can also be later translated in obesity, diabetes, and other chronic diseases and a lifetime of health issues (1–5).

Current global estimates suggest that about 10.6 million children under the age of five die annually. Of those, 3.8 million die in the first four weeks of life, with 75% occurring in the first week after birth. Neonates account for 38% of deaths in children with a similar number of stillborn (6). Most deaths occur in LIC with an estimated mortality of 33/1,000 live births, while the remaining occur in high-income countries with a clear lower rate (4/1,000 live births) (6).

Infections are the predominant cause of mortality in this vulnerable group. Major categories of neonatal deaths in poor communities include preterm birth (28%), sepsis/pneumonia (26%), tetanus (7%), diarrhea (3%), complications of asphyxia (23%), or congenital abnormalities (7%) (6). It has been described that the risk of neonatal death secondary to infections in countries with a very high-mortality is about 11-fold higher than in low-mortality countries. According to the 2020 report of the United Nations Children's Fund (UNICEF), World Health Organization (WHO), World Bank Group, and Population Division of the Department of Economic and Social Affairs of the United Nations, nearly 2 million fetal deaths occur in the world every year: with one death every 16 s, almost 4 every minute, more than 200 per hour, almost 5,400 every day, and about 164,000 every month.

In 2019, 39 countries had a higher fetal mortality rate than neonatal deaths, and 15 countries had a higher number of stillbirths than children under one year of age. Nearly 40% of global fetal deaths occur during childbirth due to poor quality of care during pregnancy and delivery (7). It is expected that an additional excess of stillbirths in low-middle income countries (LMIC) will occur due to the COVID-19 pandemic. The major social determinants affecting the under 5 years' morbidity and mortality include poverty, malnutrition, inequity, lack of education, failure to implement breast-feeding or complementary feeding programs, complications of labor and low birth weight, infections, and inadequate health-related social practices (6).

Little information is available on the aspects affecting this critical period of life in the region. Therefore, the Latin American Pediatric Infectious Diseases Society (SLIPE) convened a panel of experts to analyze the context and main barriers negatively impacting adequate development of childhood during the first 1,000 days of life and the maternal infant morbidity and mortality in LAC.

## 2. Current state of early pediatric mortality in the region

By 2020, LAC had a population of about 30,150,000 children under 2 years of age and an infant mortality rate of 14.8/1,000 live births. This means that in our region, 115,816 children under the age of one die (7). The corresponding infant mortality rates by LAC subregions, Mexico and Brazil are shown in Table 1.

**Table 1:** Infant mortality rates by Latin American subregion, Mexico, and Brazil, and (in parenthesis) specific countries with values above the regional average.

Country/Subregion	Deaths per 1,000 live births
Mexico	12.1
Central America	17.0 (Guatemala 20.0, Honduras 22.8)
Latin Carribean	34.0 (Haiti 59.0%, Dominical Republic 22.8%
Andean Area	15.8 (Bolivia 24.0, Colombia 16.8, Peru 15.0, and Venezuela 15.2)
South Cone	9.2 (Paraguay 12.6)
Brazil	13.4
Non-Latin Caribbean	17.5 (Guyana 18.9, Anguila 20.0, and Jamaica 20.9)

PAHO, Institutional Repository for Information Sharing. Indicadores básicos 2019. Tendencias de la salud en Las Américas, PCS/OMS 2019. Available at: https://iris.paho.org/handle/10665.2/51543.

**Figure 1:**
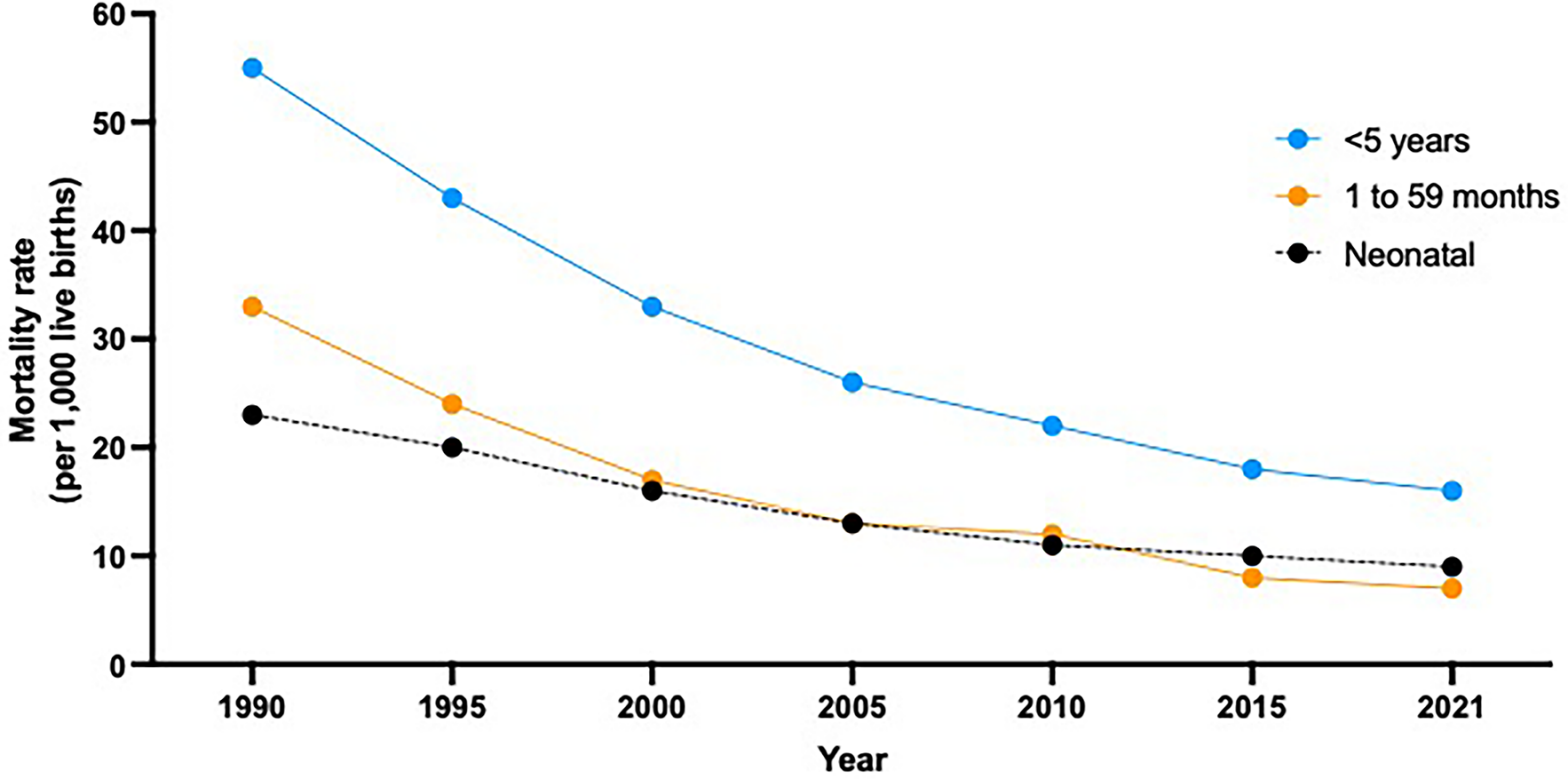
Comparative level and trends in pediatric death rates by age group (less than 5 year-old, neonates and 1 to 59 months). In Latin America and the Caribbean from 1990 to 2021. Source: Reliefweb. Levels & Trends in Child Mortality: Report 2022. Estimates developed by the United Nations Inter-agency Group for Child Mortality Estimation. https://reliefweb.int/report/world/levels-trends-child-mortality-report-2022.

The neonatal mortality rate for the region of the Americas by 2019 was 7.7/1,000 live births, and 9.3/1,000 children aged less than 28 days for the LAC region. While the neonatal mortality rate in LAC has experienced a steady decline (23 in 1990 to 9/1,000 in 2020), the global mortality in children under five years of age has fell more rapidly than the one observed in neonates (Figure 1). An estimate of 92,000 stillbirths were reported that year in LAC, that varies by country in the region: from 13.1/1,000 in Guyana, to 12.7/1,000 in Guatemala, 4.5/1,000 in Costa Rica, and 3.1/1,000 in Chile (8).

The first cause of neonatal death in the region is premature birth, followed by congenital defects, neonatal asphyxia, and trauma. Also sexually transmitted infections (STI), or respiratory tract infections account as causes of neonatal demise. During the first 28 days of life one fifth (20,000) of them are due to congenital defects (8, 9). More than half deaths before the age of five occur during pregnancy, birth, or the first four weeks of life (8, 9).

## 3. Development of immune system in early infancy

Infectious diseases represent the most frequent cause of death among infants and young children (10–16). Susceptibility to invasive pathogens is related to the fact that immune response in infants is generally less competent than in adults (17–25). Elucidating why infant immune responses to microbes and vaccines are less protective than the one observed in adults, has major implications for developing improved vaccines.

Infants display cytokine response patterns to Toll-like receptors (TLRs) stimulation that are different from those of older children and adults (26). The recent availability of system analysis tools has facilitated in depth analysis of immune responses in individuals with acute infections and in response to vaccinations. Those studies have described “immune signatures” that can let the identification of early gene expression patterns and cell populations that correlate with, or even predict, the development of protective immune responses (27–31).

Increasing evidence indicates that the initial encounter with a specific antigen or pathogen, whether it is in the context of infection or vaccination, has profound implications for future immune responses. The best-known example is influenza virus where initial exposure to the virus via infection and/or vaccination shapes the establishment of B cell memory and the responses to subsequent infections and vaccinations, a process known as the “original antigenic sin” (32). It has been proposed that this phenomenon of “immune imprinting” in not unique to influenza virus and that it may also occur in the context of other viral infections such as coronaviruses or dengue virus (33, 34).

There is a concept that infants have predominantly naïve B and T cells, and the numbers of memory B and T cells increase progressively as they get exposed to different pathogens and vaccines. For this reason, in the initial months of life infants rely mostly on maternal antibodies and the innate immune responses. However, these notions are being challenged by recent data. Analysis of young infants infected with Respiratory Syncytial Virus (RSV) showed that the innate immune response, and more specifically, the antiviral interferon response is not very effective during the first 6 months of life (34, 35). Indeed, this inadequate interferon response has been associated with poorer clinical outcomes, which may explain in part why RSV causes such severe disease, especially in the first months of life (36).

Animal studies suggest a potential role of the microbiome composition in modulating responses to vaccines (37). A recent retrospective study provided evidence that young children to 24 months of age who were prescribed antibiotics had a higher frequency of vaccine-induced antibody titers below the levels of protection compared with children with no antibiotic prescriptions. Furthermore, investigators observed a dose-effect as the total antibiotic exposure was associated to lower antibody titers. The authors proposed that antibiotic treatment in early life modified the gut microbiome, which in turn altered immune responses to routine vaccines (38). Despite the limitations of the study design, these provocative observations clearly deserve future prospective studies as their findings could have major repercussions for the immunization programs (39).

These are a few examples of how the unique features of the infant immune system significantly impact childhood health and have long-term consequences for establishing protective immunity. For these reasons, and despite the challenges involved in conducting clinical studies in young children, it is essential we dedicate renewed efforts to improve our understanding of early life immunity to further develop more effective vaccines and therapies for children.

## 4. Determinants that affect child's health from preconception to two years of life

### 4.1. Maternal health

An essential element of the first 1,000 days of life is the health condition of the pregnant woman, not only along pregnancy, but also during childbirth and puerperium. High maternal mortality rates may be related to poor reproductive health, including lack of access to proper care during pregnancy and childbirth, and availability of safe abortion whether legal or not. Poverty, low economic status, lack of education, poor nutrition, heavy workloads, and violence may increase the risks of poor outcomes during pregnancy and childbirth (40). According to the State of the World's Children 2023 report from UNICEF, they all are strongly associated with poor vaccination coverage rates in the region (40).

Maternal mortality is defined as the death of a woman during pregnancy, childbirth, or the first 42 days after delivery. In the last decade, LAC have shown modest reduction in maternal mortality, however, the COVID-19 pandemic, has increased the number of deaths reported (41). The greatest risk of maternal mortality is age, and it includes adolescents under the age of 15 in most developing countries. Severe hemorrhages, infections, complicated hypertension (preeclampsia and eclampsia), and dangerous abortions account for 75% of maternal deaths (41).

LAC reported a maternal mortality rate of 74/100,000 live births in 2017, and by 2020, it decreased to 67.2/100,000. According to the Sustainable Development Goal (SDG), this rate should be lower than 70/100,000 live births for 2030 (41, 42), but there are still several countries in the region with higher mortality index.

### 4.2. Food security and nutrition

The 2021 United Nations report on regional food and nutrition security reported that LAC experienced a 30% increase in hunger between 2019 and 2020. About 4.8 million boys and girls under five years of age in the region are affected by chronic malnutrition (43). Over one year, in the context of the COVID-19 pandemic, the number of people living with hunger increased by 13.8 million, reaching a total of 59.7 million people (43).

The prevalence of hunger in LAC is currently 9.1%, the highest of the last 15 years. It has been estimated that four out of ten people in the region (about 267 million) experienced moderate to severe food insecurity in 2020; that is, 60 million more than in 2019, mainly in Mesoamerica (43, 44). By 2020, the malnourished population reached 16.1% in the Caribbean, 10.6% in Mesoamerica, and 7.8% in South America (43, 44).

Maternal food deprivement is also related to other conditions. The region has not shown significant progress in reducing the prevalence of anemia in women of reproductive age, that by 2019 reached 17.2%. There is a goal set by the SDG-2 to reduce anemia in 50% by 2030, not yet accomplished in LAC. Nowadays anemia affects 14.6% of women in reproductive age in Mesoamerica, 17.3% in South America, and 29.2% in the Caribbean. Countries with the highest prevalence in the region are Haiti (47.7%), Guyana (31.7%), and Dominican Republic (26.4%), in contrast with countries such as Chile (8.7%) and Guatemala (7.4%) (43).

Regional rate of exclusive breastfeeding during the first six months of life is 33.4%, much lower than the world's average (44%) (45). This may be related with children under five years of age with failure to thrive in LAC, an indicator of disease, poor health, and malnutrition, that ranges between 7.3%–12.6% in 2019 (46). All these factors increase the risk of complicated infections and death during the first 1,000 days of life.

### 4.3. Exposure to toxins, pollutants, and infectious agents

Exposure to toxic chemicals can lead to chronic and often irreversible health disorders, including birth defects, neurodevelopmental delay, and endocrine disrupted diseases. Chemical hazards, such as exposure to pesticides, lead, and mercury, tend to disproportionately affect children and pregnant women. Pediatric population, especially young children, are exposed to continuous pollutants, increasing the risk of premature deaths and diseases related to multiple sources of contamination.

In the Americas, about 847,000 deaths per year (13%) are attributable to environmental risks. Air pollution is the main public health hazard in the Americas, where more than 150 million people in LAC live in cities that exceed the WHO Air Quality Guidelines. Consequently, 320,000 preventable deaths in the Americas result from air pollution (47).

Water pollution is also an important problem. Twenty-eight million people in the region lack potable water access, and 83 million lack adequate basic sanitation services and facilities, causing nearly 30,000 preventable infectious related deaths. Approximately 7,600 children under five years of age die annually from acute diarrhea in the region. Haiti (23%), Guatemala (10%), Bolivia (7%), and Venezuela (5%) have the highest rates of mortality (8).

Children are particularly susceptible to the harmful effects of the environment. The impact of heavy metals such as lead and mercury on the pregnant woman and the newborn brings harmful consequences for children development, cognitive capacity, and growth. Those under five years of age are particularly vulnerable because depending on the source of contamination, they absorb 4–5 times more lead than adults (48, 49). Harmful effects of lead include premature birth, low birth weight, developmental delay, learning difficulties, and growth retardation in young children. These effects are more common in risk exposures from the mother or the baby (49, 50).

It is estimated that in LAC 49,107,507 children between 0 and 9 years of age have blood levels of lead above 5 ug/dl. Elevated lead levels in children have been reported in Mexico, Colombia, Peru, and Brazil (50). Countries of the Amazon basin (Brazil, Colombia, Peru, Ecuador, Venezuela, and Bolivia) have reported high concentrations of mercury in children, including newborns, detected in the umbilical cord and breast milk (49). In Latin America, more than 4.5 million people are chronically exposed to arsenic levels in drinking water, more than 200 times higher than the WHO limit (10 *μ*g/L). In a systematic review conducted by Heng et al., it was shown that postnatal exposure to lead and manganese is associated with neurodevelopmental delay (48, 49).

Exposure to lesser common toxins is also a health menace. More than 80 million people still depend on polluting fuels such as solid fuels or kerosene for lighting, cooking, and heating (47).

As mentioned before, infectious disease account for an important risk factor of death in the first 1,000 years of life. According to recent data, it is estimated that approximately 3% of the deaths in young children are caused by meningitis (51). The most common bacteria causing meningitis include *Streptococcus agalactiae*, enteric bacilli, occasionally *Listeria monocytogenes* during the first two months of life, *Streptococcus pneumoniae, Neisseria meningitidis,* and *Haemophilus influenzae*, but their relative contribution differs over time, region, and age group (52, 53). In Latin America, as well as in other LMIC countries, tuberculosis is also a relevant cause of meningitis in young children (51).

Most of the clinical burden of acute gastroenteritis occurs in young children, mainly in developing countries (54). Studies carried out in our region have revealed the etiological importance of rotavirus and norovirus, that represent almost half of hospitalizations during early childhood. Other organisms described are *Shigella sp.*, *Giardia sp.*, *Campylobacter sp.*, and pathogenic strains of *Escherichia coli* (55, 56).

Acute respiratory infections are also a leading causes of child morbidity and mortality, causing close to one third of deaths in children under five years of age in LIC (57, 58). In the WHO Region of the Americas report, pneumonia and influenza accounted for 11.5% of all deaths in children 0–4 years of age in 2007. Typical bacterial pathogens that cause community acquired pneumonia are *S. pneumoniae, H. influenzae* (both vaccine-preventable infections), *Mycoplasma pneumoniae,* and *S. aureus*. In Latin America, the predominant infections are caused by *S. pneumoniae*, *H. Influenzae* type b, Respiratory Syncytial Virus, Influenza virus, and recently, SARS-CoV-2 (57, 58).

### 4.4. Migration and climate change

Migrant children and pregnant women are affected by social and health inequities. This vulnerable population is also exposed to violence, sexual exploitation, and sexually transmitted diseases. Venezuelan migration within the Americas has accounted for 3.9 million people moving from their country, where 24% were children (59, 60).

Vulnerable groups are disproportionately at risk from increased frequency and severity of extreme weather conditions (60). Climate-related factors have an impact on health and well-being of population because they disrupt the world's physical, biological, and ecological systems. Health effects may include, but are not limited to, increased respiratory and cardiovascular diseases, injuries and premature deaths related to extreme weather events, food insecurity and air pollution, threats to mental health, and changing patterns of vector-borne diseases transmission and climate-sensitive infections (60).

The SDGs for 2030 Agenda addresses the environmental determinants of health and contribute directly and indirectly to SDG-3 pertaining “Good Health and Well-being” to ensure healthy lives and promote well-being for all. Issues include water supply, sanitation and hygiene, air quality, chemical safety, and climate action (61).

## 5. SLIPE advisory group recommendations

The Advisory Group of SLIPE has defined ten general strategies aimed to reduce the maternal and infant mortality associated with infections and immune preventable diseases during the first 1,000 days of life in LAC.

### 5.1. Problem awareness

It is necessary to increase awareness in the medical and public community about the determinants of maternal and infant morbidity and mortality in the region. Provide universal access to medical care with a focus on vulnerable populations in LAC, such as mothers and children, is essential. Health policies are necessary to reduce this burden, and improve food and nutritional security, reduce the exposure to infectious agents, and provide adequate healthcare to populations are affected by natural phenomena caused by climate change.

### 5.2. Universal access to mother's health

One of the most effective means to improve maternal health is to invest in primary health care and improve health systems to ensure availability and professional care at all levels. Family planning services, prevention of unplanned pregnancies, and access to safe abortion and post-abortion care services may significantly impact maternal deaths and morbidities (40).

Preventive health assessments for women before and during pregnancy play a crucial role in reducing the risk of adverse health effects for both the women and their infants. While prenatal care is generally elevated in LAC (62), several other aspects of health require attention. These include the management of chronic conditions, the appropriate use of prescribed and non-prescribed medications, nutrition evaluation, screening for violence, assessing security and living conditions, ensuring adequate support postpartum, and addressing mental health. In some settings, these aspects are not always prioritized for pregnant women (63).

It's important to recognize that safeguarding the health of newborns begins with the health of pregnant women, and assessing vaccination status during prenatal care should be a standard practice in every health intervention (63).

### 5.3. Prevention of climate change and environmental impact on health

Environmental public health programs must assess health issues attributable to climate factors trough the development of inclusive and equitable policies to protect people from environmental hazards. This is achieved through pragmatic, intersectoral, multisectoral, subnational, national, and supranational approaches. It is important that public health programs foster an environmentally responsible and resilient health sector.

To reduce the burden of disease and health inequity attributable to environmental determinants of health in the region is necessary to increase the capacity of health actors and to address the environmental determinants of health, emphasizing air quality, safety of chemical substances, factors associated with climate and water, sanitation, and hygiene, prioritizing populations living in vulnerable situations.

Actions must be focus on improvement of public health programs, environmental public health surveillance, and promotion of responsible and resilient health sector.

### 5.4. Access to food security and nutrition in LAC

Optimal physiological, cognitive, and emotional development in children requires access to food of quality and in quantity at this critical period. Unfortunately, according to the United Nations report “Overview of Food Security and Nutrition 2022”, hunger in LAC reached 56.5 million in 2021, with the Caribbean as the most affected area (64). By 2021, 40.6% of population experienced moderate to severe food insecurity, compared to 29.3% worldwide (64).

A worrisome amount of 131.3 million people in the region were not able to afford a healthy diet in 2020, largely due to costs as compared to other regions. This situation affects more severely vulnerable populations such as small farmers and women in rural areas, as well as indigenous and Afro-descendant populations. The average cost of a daily healthy diet in the Caribbean has been estimated in USD 4.23, followed by USD 3.61 in South America, and USD 3.47 in Mesoamerica (64). While no individual policy can solve this problem, regional governments need to develop public policies that guarantee adequate nutrition and availability of nutritious food at affordable prices, in addition to nutritional counseling, in vulnerable communities.

Food insecurity may be both a driver and an effect of migration. While incidental evidence indicates that it is common to identify food insecurity in migrants, is not well documented. Both migration and food security are complex events, and migration presents both challenges and opportunities in food security, as they are heavily influenced by local conflicts, natural threats or disasters, climate change, and poverty, but they are also a vital component of the work force of the food processing industry and contribute to food security through agricultural labor (65).

### 5.5. Implementation of general measures to promote a healthy parenthood: availability of adequate infectious diseases evaluation at pre-conception and during pregnancy. Specialized care during childbirth, adoption of infection prevention practices during pregnancy and early postpartum

Elimination of mother-to-child transmitted diseases is a priority in the region of the Americas. Work should begin prior to pregnancy among adolescents and women of childbearing age, with special focus on the most vulnerable segments of the population, through counseling, prevention of sexually transmitted infections, contraception, comprehensive sexual health clinics, and counseling on pre- and post-exposure HIV prophylaxis.

Difficulties accessing health services and vulnerabilities of groups are fundamental factors conditioning the analysis of the interventions to be carried out. Emphasis must be placed in avoiding unplanned pregnancies and the implementation of measures to improve health conditions of each woman prior to the beginning of pregnancy.

Access to appropriate prenatal care, with specialized attention to childbirth and immediate postpartum should be guaranteed, including measures aimed to generate practices to prevent infections during pregnancy and in a hospital setting. Infections acquired prior to pregnancy, during pregnancy or peripartum, can affect maternal health, cause pregnancy loss, intrauterine growth restriction, neonatal deaths, and a wide variety of sequelae that can manifest throughout life.

Investigation of asymptomatic infections and identification of risk situations will allow the application of preventive or therapeutic measures to reduce maternal, fetal, and neonatal morbidity and mortality.

Strategies aimed to the community and hospitalized women of child-bearing age are necessary to encourage responsible procreation, prevention of sexually transmitted infections, and infections associated with health care. Joint work of obstetricians, pediatricians, neonatologists, biochemists, nurses, infectious diseases specialists, and health agents is vital.

### 5.6. Appropriate fulfillment of maternal immunization schedules before and during pregnancy

During pregnancy, up-to-date routine immunizations should be guaranteed, and previous immunization schedules should be reviewed, as some vaccines cannot be offered in a gestated woman, but disease may cause severe birth defects (66–68). Everyone in the household must be up to date with their immunizations, lowering the chance of vaccine preventable diseases in pregnant woman.

#### 5.6.1. Recommended vaccines for pregnant women

Pregnant women are advised to receive influenza vaccine at any time during pregnancy, pertussis-containing vaccines (Tdap) between second and third trimester (Table 2). SARS-CoV-2 infection during pregnancy has been associated with a higher risk of preterm birth and stillbirth. Therefore, health authorities in LAC and North America recommend COVID-19 vaccination for everyone aged 6 months and older, including people who are pregnant, breastfeeding, or might become pregnant in the future. This recommendation includes getting boosters as recommended (69).

**Table 2:** Recommended vaccines for pregnant women.

Vaccine	Recommendation	Comments
Inactivated influenza	Recommended for all pregnant women at any stage of pregnancy, especially in the second or third trimester in countries with influenza season	Pregnancy increases the risk of severe influenza. Influenza immunization protects the mother, and the newborn baby during the first few months after birth. Clinical trial data and observational studies show no increased risk of congenital defects or adverse effects to the fetuses of women who received influenza vaccine during pregnancy.
Tdap (diphtheria-tetanusacellular pertussis) Td (diphtheria-tetanus)	Recommended as a single dose between the mid second trimester and early third trimester of each pregnancy (ideally from week 20 to 32)	Vaccination during pregnancy reduces the risk of pertussis in pregnant women and their young infants by about 90%. Available data show no evidence of increased risk of adverse pregnancy outcomes related to pertussis vaccination during pregnancy.
		Td is also recommended to prevent maternal neonatal tetanus (MNT).
COVID-19	Recommended for all pregnant women at any stage of pregnancy	Pregnant women are more likely to be hospitalized and die from severe COVID-19, and the disease puts them at greater risk for preterm birth.
		Vaccine induce protective specific antibody titers both in the mother and in the newborns immediately after birth'a critical time when children are vulnerable to severe COVID-19 disease but are too young to be vaccinated, according to the researchers

Pregnant women can recieve Td or Tdap vaccines for the prevention of maternal-neonatal tetanus, or to manage tetanus-prone wounds. Also, they can receive rabies vaccine if required for post-exposure prophylaxis, with a significant risk of exposure (related to occupation or travel), or if there has been a potential exposure to rabies virus or another bat lyssavirus (70, 71).

Live attenuated vaccines are generally contraindicated in pregnant women due to a hypothetical risk of harm if the vaccine pathogen replicates in the fetus (66–68). Although pregnant women are not recommended to receive yellow fever vaccine, travel to a country with risk of yellow fever transmission, or to endemic areas may be unavoidable. In this case, risks and benefits of yellow fever vaccination must be weighted, and other strategies to reduce risk of infection should be contemplated (66–68).

#### 5.6.2. Immunization of breastfeeding women

Vaccination is rarely contraindicated in breastfeeding women. For most vaccines, an infant's immune response to vaccination in relation to breastfeeding has been considered. In general, breastfeeding does not adversely affect immunization, and is not a contraindication for any vaccines recommended in infants.

Breastfeeding women should not receive yellow fever vaccine unless the mother has a high risk of acquiring yellow fever or cannot avoid or postpone travel. Although extremely rare, there have been case reports of probable transmission of the yellow fever vaccine virus to infants through breast milk (72, 73).

### 5.7. Completion of vaccine calendars during the first and second year of life, according to national vaccination programs

Vaccines in children from the neonatal period until the age of 24 months helps to protect them from serious and life-threatening diseases. Early vaccination can help ensure children's protection when they are most at risk. Additionally, it may help prevent the spread of these diseases within communities, providing protection not only for the vaccinated child but also for those who are too young or unable to receive vaccines themselves through herd immunity.

Recommended vaccines from birth to 24 months of age may vary in every region. Globally, twenty-five new vaccine introductions were recorded in 2021 (not including COVID-19 vaccine). Although this is an increase from 2020, it is well below the number of introductions of any year in the past two decades prior to 2020. This slowdown is likely to continue as countries focus on ongoing efforts to control COVID-19 pandemic (74).

While immunizations have been one of the most successful interventions to reduce or eliminate the incidence of vaccine-preventable diseases, vaccination coverage has decreased over the years, even before COVID-19 (75). While the pandemic did cause a frightening drop in coverage due to intrinsic conditions of social isolation widely discussed in different publications, regional trends in vaccination coverage in Latin America had already experienced a progressive reduction that intensified in 2020 (76).

In 2021, more than 2.7 million children under one year of age in the Americas (19.7%) did not receive all their shots, leaving them susceptible to diseases such as polio, tetanus, measles, and diphtheria. Two years into the pandemic, health services have not yet fully recovered (75, 76).

**Figure 2:**
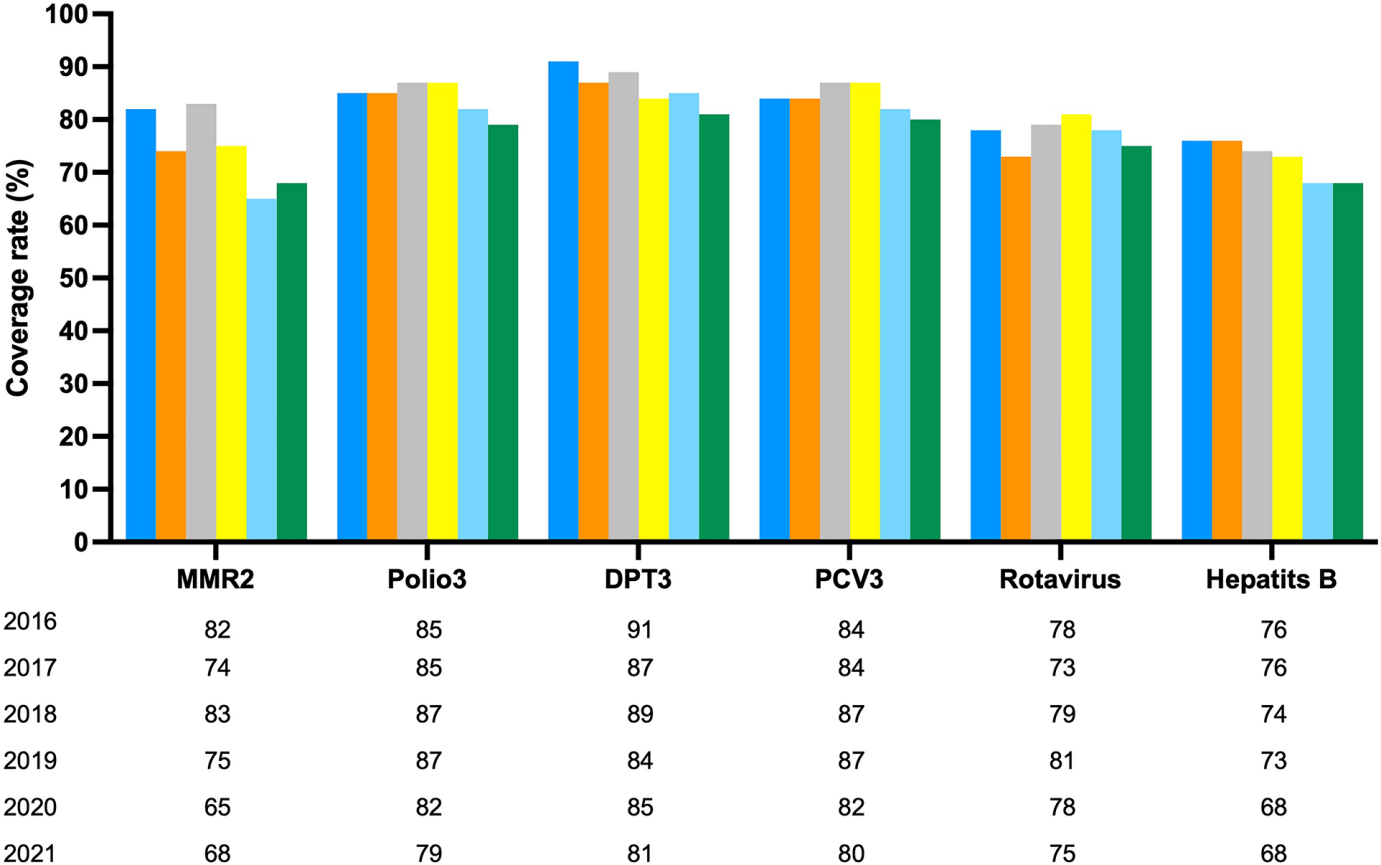
Vaccination coverage of selected vaccines in region of the Americas, 2016–2021. Country reports through the PAHO-WHO/UNICEF Joint Reporting. Source: IRF and country submissions of the electronic. PAHO-WHO/UNICEF Joint reporting From (e.IRF). Preliminary data as of 04 August 2022.

As seen in Figure 2, regional vaccination coverage rates for children have shown a steady percentual decline since 2016, including MMR (17.08%), polio (7.06%), DPT (11.09%), PCV (4.77%), rotavirus (3.85%), and hepatitis B (10.53%) (74–76).

Over the last decade, overall vaccination rates for children in the continent fell from 93% to barely 75%, which places LAC below the world average (81%) with regions such as the East and Southern Africa (74%). Moreover, by 2023, more than 1.7 million children in the region had not received any dose of any vaccine, a phenomenon known as “zero children”. The most affected countries are Brazil (700,000), Mexico (316,000), and Venezuela (120,000). These three countries account for more than 60% of unimmunized minors in the region. Brazil and Mexico also appear on the list of the 20 countries with the higher number of unvaccinated children. Main causes include migration, natural disasters, political instability, and violence. Children in situation of poverty are three times more likely to be in this group. This vulnerable population is increasing in number, partially because immunization is lower on the list of priorities for families and countries (74–76).

Vaccine hesitancy has been stable in the region until the beginning of the pandemic, that negatively impacted vaccination coverage rates (77). Rejection has been mainly identified towards mRNA vaccines, most importantly in the group below 5 years of age. COVID-19 brought a negative impact on national vaccination programs secondary to a decrease in the demand during confinement, decrease capacity for epidemiological surveillance, and weakness of health systems around the region (77).

Despite progress in vaccination, collective efforts have fallen short. While new vaccines are being developed or are in the pipeline, achieving sufficient coverage remains a challenge. Ensuring the right of all children to be immunized involves comprehensive programs with adequate infrastructure and resources, education of healthcare workers and communities about the importance of immunization, and collaboration with national entities and international organizations (40).

Countries need to strengthen their national health systems to provide universal coverage, making all available vaccines accessible to every child. Despite the rapid development of vaccines during the pandemic, global distribution and uptake remained inadequate (78).

Equity in immunization should be a priority, viewing it as a lifesaving intervention. The goal is to vaccinate every child, everywhere, build confidence in vaccination, invest in vaccines, and fortifying national immunization programs through resilient strategies. This approach aligns with the principle that access to vaccines is a fundamental right for every child, regardless of geographical location or socio-economic status (40).

### 5.8. Prompt clinical evaluation of newborns with suspicion of congenital infection under standardized diagnostic and treatment guidelines

LAC is a region with high prevalence of syphilis and HIV. The elimination of Mother-To-Child Transmission (MTCT) of these diseases is a priority in the region (62). A 10% maternal transmission rate of HIV, and 2 cases of congenital syphilis (CS) per 1,000 live births are estimated, with important differences between different countries (0–6.5/1,000 live births in 2015). Significant progress has been made towards the goal of disease eradication, and eight countries in the region have achieved certification of dual elimination of HIV and CS. Nevertheless, increased efforts are needed to achieve greater accessibility in the evaluation in pregnant women and their partners including optimization of the frequency and quality of follow up appointments during pregnancy, accessible education for sexually transmitted diseases prevention, adequate treatment for the pregnant woman and her/his/their partners, and improved epidemiological reporting systems to achieve sustained control of these infections (79).

In September 2010, the PAHO/WHO approved the strategy and Plan of Action for the elimination of MTCT of HIV and CS, with the goal of reducing MTCT of HIV to 2% or less, and the incidence of CS to 0.5 cases (including stillbirths) or less per 1,000 live births by the year 2015 (80). This commitment was renewed and expanded in 2016, when the member states approved the “Action Plan for the Prevention and Control of HIV Infection and Sexually Transmitted Infections (STIs) 2016–2021” or ETMI-Plus incorporating Hepatitis B and Chagas into the disease eradication strategy (79).

*Streptococcus agalactiae* or beta-hemolytic Group B (SGB) continues to be one of the main pathogens of early neonatal sepsis (ENS). The detection of the maternal colonization by SGB and the intrapartum antibiotic prophylaxis have managed to reduce by nearly 75% cases of ENS. It's key to implement in our countries policies that allow the identification of pregnant women through vaginal and rectal cultures to search for SGB after 35 weeks of gestational age (81).

There are no worldwide standardized recommendations over the prevention of congenital toxoplasmosis. In Latin America, Colombia, Argentina, Uruguay, Paraguay, Brazil, Panama, and other countries there is a proposal for a screening program, but this isn't the case in other countries like Chile, Peru, Mexico, and Ecuador. Several studies have been performed in both France and Colombia that show that cost benefit of screening, even in locations with few cases (82, 83).

The introduction of an investigation protocol (before conception and labor), the diagnosis, the timely treatment during pregnancy, and the evaluation and monitoring of newborns has diminished cases of children with severe sequelae due to congenital toxoplasmosis (84, 85).

Cytomegalovirus (CMV) is the most frequent reason for congenital infections. The fetal infection can occur due to a primary maternal infection during pregnancy or secondarily through a viral reactivation or reinfection. Hearing loss is the main consequence of congenital CMV and the leading cause of non-genetic deafness among children (86). There are no recommendations of serological studies for CMV nor of effective treatments for pregnant women with a primary infection or reinfection. Several screening programs for newborns are currently undergoing with the goal of diagnosing and treating congenital infection at an early stage and prevent the main hearing and neurological consequences that might develop (87).

The main source of infection for CMV during pregnancy is contact with children under five years old which are in the early stages of their education. Apart from the serological results for CMV, pregnant women must be warned and educated about the potential risk of infection that contact with the saliva and urine of children brings, and they must be reminded of how important it is to wash their hands (88).

All newborns, regardless of their clinical condition, must be evaluated to rule out congenital infections. The evaluation of both the mother and child in the first days of neonatal life offers the opportunity to analyze the maternal clinical history during pregnancy, establish epidemiological determinants of risk, and review compliance and results of the serological tests recommended in each region. If testing is incomplete, this approach provides the opportunity to complete them prior to discharge.

Children who present with clinical stigmata suspicious for congenital infections should be evaluated by first reviewing maternal history and serological results, ranking the most frequent infections.

#### 5.8.1. Need to elaborate consensus guidelines to detect risks in children with fever in the first year of life

Fever represents one of the main reasons for pediatric consultation in the first 1,000 days of life. For different reasons children have a greater susceptibility to viral and bacterial infections, which favors the use of antibiotics.

To prevent the excessive use of antibiotics without neglecting the possibility of a serious hidden bacterial infection, risk criteria and diagnostic algorithms for the clinical management of febrile children have been established (89, 90). In addition, with the universal introduction in many sites of effective conjugate vaccines and the incorporation of new molecular tests into the laboratory, documenting a viral etiology has become particularly relevant, especially in the era of antibiotic resistance.

The neonatal period is certainly one of the most important in the management of the pediatric patient with fever and the one with the highest risk for serious bacterial infections. The febrile neonate must be carefully evaluated and, except in very rare cases, must be hospitalized and treated with antibiotics while the pertinent tests are performed to rule out early or late onset sepsis. Ideally, the mother should have been screened for the presence of vaginal and/or rectal colonization by *Streptococcus agalactiae* (Group B) during pregnancy (89–91).

Beyond the neonatal period and during the first 1,000 days, other risk factors for severe infection become significant, such as protein-calorie malnutrition, lack of breastfeeding, incomplete vaccination schedule, immunosuppressive treatment or immunodeficiency, environmental health conditions, and the presence of infections in the child's home environment (89, 90).

Because of the widespread use of conjugate vaccines, the febrile child is much less likely to have an invasive bacterial infection from the encapsulated pneumococcus, meningococcus, and *Haemophilus influenzae* type b (90). *Escherichia coli* urinary tract infections or staphylococcal infections tend to prevail. To guarantee the well-being of young children, it is important to implement clear diagnosis and treatment guidelines in our region to detect viral pathogens, as well as to suspect the presence of serious infections and guarantee the adequate use of antibiotics (10, 89, 90). This would contribute to reduce antimicrobial resistance, a growing problem that must be faced wisely and responsibly.

The coexistence of differing clinical strategies for the management of young febrile infants from various national professional societies or organizations with broad representation, underlies the need for an evidence-based guideline adapted to the realities of our region.

### 5.9. Encouragement of optimal breastfeeding practices to decrease child death and contribute to the long-term health of children

Multiple studies have shown that breast milk decreases infant mortality. Non-breastfed infants may suffer from more frequent and severe infectious diseases, with a risk of hospitalization up to seven times higher than those receiving breastfeeding (92). Breast milk has all the necessary nutrients for the first months of life: fats, carbohydrates, proteins, vitamins, and minerals. It also contains bioactive factors such as enzymes, hormones, immunomodulatory factors, and bifidogenic components that, in combination with leukocytes, prevent the adherence infectious agents in the neonate and infant. In addition to other nutritious, immunological and emotional advantages, the WHO recommends exclusive breastfeeding until 6 months and supplemented until 2 years of age (92). Unfortunately, the data available from the LAC region reveal that only 50% of newborns are breastfed within the first hour of birth and only 37% maintain continuous breastfeeding during the first 6 months (92, 93).

Breastfeeding allows transfer of immunocompetence to the newborn and the subsequent maturation of the immune system for protection against microbes (94). Human milk also contains lactoferrin, a substance with antimicrobial properties and the ability to modulate the innate and adaptive immune response (95). Its antimicrobial capacity is based on the chelating action of iron necessary for pathogens, which provides bacteriostatic and bactericidal power. The hydrolysis of lactoferrin by pepsin gives rise to peptides called lactoferrin H and B that have greater antimicrobial potency than the predecessor molecule. Other peptides with antimicrobial action are components derived from lactalbumin, lactoperoxidase, lysozyme, milk mucins, tenascin (glycoprotein), and bioactive peptides capable of inhibiting viral growth or suppressing microbial replication (96–98).

Studies have shown a variety of benefits of breastfeeding, of which the decrease in necrotizing enterocolitis, late sepsis, retinopathy of prematurity, and rehospitalization in the first year of life, stand out. More recently, studies have delved into the composition of human breastfeed, and have also established differences between mothers of premature and term infants. In addition, lactoferrin is higher in premature mothers and more concentrated in colostrum (8, 9).

A review to determine the impact of breastfeeding on the rate of hospitalizations in healthy, full-term infants younger than 8 months of age, showed that exclusive and prolonged breastfeeding is a protective factor for hospital admission due to diarrhea or lower respiratory tract infection. However, the protective effect of breastfeeding disappears as time progresses (8). In a prospective cohort study conducted in Turkey to analyze the impact of breastfeeding on frequent pediatric infections, a significant decrease in otitis media and gastroenteritis during the first five years of life with breastfeeding for more than 12 months, was found. These infections were significantly lower in children who had an exclusive breastfeeding for 6 months (40).

Another potential protective effect of breastfeeding is the stimulation of growth of non-pathogenic flora, which reduces colonization by enteropathogens, improves the development of mucosal barriers, and facilitates passage of immune cells, proteins, and anti-infectious enzymes (99). The microbiota present in milk also has peptides and lipids with antimicrobial activity (100).

Protection against some pathogens in the neonatal period can be obtained with the passive acquisition of maternal antibodies. It is well known that the secretory antibodies of human milk from vaccinated pregnant women, such as IgA, the immunoglobin predominant in human milk, protect against influenza (101, 102). The transfer of antibodies into the breast milk of lactating women one week after the initial dose of COVID-19 mRNA vaccine has been demonstrated, suggesting the possibility of passive protection for the infant after maternal vaccination (103).

It has been proposed that exclusive breastfeeding for the first 6 months of life, is the preventive intervention that has the greatest potential impact on infant mortality (104). It does not only provide the necessary and unique nutrients for growth and development, but also becomes the first immunization for the infant (105).

### 5.10. Rationale and prudent use of antibiotics

Antimicrobial resistance is one of the greatest public health challenges of our time since few treatment options exist for people infected with drug-resistant bacteria. Whereas antibiotics may be lifesavers, they can also be associate with unnecessary adverse events and contribute to the occurrence of antimicrobial resistance.

Animal studies suggest a potential role of the microbiome composition in modulating responses to vaccines (37). A recent retrospective study provided evidence that young children to 24 months of age who were prescribed antibiotics, had a higher frequency of vaccine-induced antibody titers below the levels of protection compared with children with no antibiotic prescriptions. Furthermore, investigators observed a dose-effect between total antibiotic exposure and lower antibody titers. Antibiotic treatment in early life might modify gut microbiome, alter immune responses, and affect vaccine effect (38). These provocative observations clearly deserve future prospective studies as their findings could have major repercussions for immunization programs (39).

Smart use of antibiotics is a key part of best care. Prescribing antibiotics only when needed helps fight antibiotic resistance, and ensures that these life-saving drugs, will be available for future generations (106).

## 6. Conclusions

LAC still faces immense challenges to overcome the barriers that negatively impact the early and healthy development of children during the first 1,000 days of life. Infectious diseases are a relevant cause of morbidity, associated with significant maternal and infant mortality in the region. Health policy makers from health organization that generate influence in Latin American governments need to be aware and plan strategies within health policies directed to reduce maternal and infant death.

The ten strategies presented by the Advisory Group of SLIPE focus on the current gaps and obstacles identified in the region that negatively affect this vital phase of life. Feasible strategies are provided gradually overcome these barriers. However, to carry out these recommendations, countries of the region need proper planification to accomplish these measures. Suitable venues to facilitate implementation of specific plans, needs to be part of a comprehensive program to optimize attention during this critical period of child's development.

## References

[1] Scott JA. The first 1000 days: a critical period of nutritional opportunity and vulnerability. Nutr Diet. (2020) 77(3):295–7. 10.1111/1747-0080.12617 32478460

[2] Lake A. The first 1,000 days of a child’s life are the most important to their development—and our economic success. World Economic Forum Annual Meeting (2017). Available at: https://www.weforum.org/agenda/2017/01/the-first-1-000-days-of-a-childs-life-are-the-most-important-to-their-development-and-our-economic-success/

[3] Walker SP Wachs TD Gardner JM Lozoff B Wasserman GA Pollitt E Child development: risk factors for adverse outcomes in developing countries. Lancet. (2007) 369(9556):145–57. 10.1016/S0140-6736(07)60076-2 17223478

[4] Chang S Zeng L Brouwer ID Kok FJ Yan H. Effect of iron deficiency Anemia in pregnancy on child mental development in rural China. Pediatrics. (2013) 131(3):e755–63. 10.1542/peds.2011-3513 23400604

[5] Black RE Allen LH Bhutta ZA Caulfield LE de Onis M Ezzati M Maternal and child undernutrition: global and regional exposures and health consequences. Lancet. (2008) 371(9608):243–60. 10.1016/S0140-6736(07)61690-0 18207566

[6] Bhutta ZAS. M. A. Childhood infectious diseases: overview. Int Encycl Public Health. (2008):620–40. 10.1016/B978-012373960-5.00568-2

[7] UNICEF. A neglected tragedy. the global burden of stillbirths (2020). Available at: https://data.unicef.org/resources/a-neglected-tragedy-stillbirth-estimates-report/

[8] Organización Panamericana de la Salud. Indicadores Básicos 2019. Tendencias De La Salud En Las Américas (2019). Available at: http://www.bvs.hn/docum/ops/IndicadoresBasicos2019_spa.pdf

[9] Banco Mundial. Tasa De Mortalidad, Neonatal (Por Cada 1.000 Nacidos Vivos)—Latin America & Caribbean (2021). Available at: https://datos.bancomundial.org/indicador/SH.DYN.NMRT?locations=ZJ

[10] Global Health Observatory, World Health Organization. Causes of child mortality for the year 2010 Who, Geneva: (2021). Available at: https://www.who.int/data/gho/data/themes/topics/topic-details/GHO/child-mortality-and-causes-of-death

[11] Chopra M Mason E Borrazzo J Campbell H Rudan I Liu L Ending of preventable deaths from pneumonia and diarrhoea: an achievable goal. Lancet. (2013) 381(9876):1499–506. 10.1016/S0140-6736(13)60319-0 23582721

[12] Nair H Simoes EA Rudan I Gessner BD Azziz-Baumgartner E Zhang JSF Global and regional burden of hospital admissions for severe acute lower respiratory infections in young children in 2010: a systematic analysis. Lancet. (2013) 381(9875):1380–90. 10.1016/S0140-6736(12)61901-1 23369797 PMC3986472

[13] Rudan I Nair H Marusic A Campbell H. Reducing mortality from childhood pneumonia and diarrhoea: the leading priority is also the greatest opportunity. J Glob Health. (2013) 3(1):010101. 10.7189/jogh.03.010101 23826497 PMC3700027

[14] Rudan I O'Brien KL Nair H Liu L Theodoratou E Qazi S Epidemiology and etiology of childhood pneumonia in 2010: estimates of incidence, severe morbidity, mortality, underlying risk factors and causative pathogens for 192 countries. J Glob Health. (2013) 3(1):010401. 10.7189/jogh.03.010401 23826505 PMC3700032

[15] Walker CLF Rudan I Liu L Nair H Theodoratou E Bhutta ZA Global burden of childhood pneumonia and diarrhoea. Lancet. (2013) 381(9875):1405–16. 10.1016/S0140-6736(13)60222-6 23582727 PMC7159282

[16] World Health Organization. Regional Update Ew 39, 2013: Influenza and Other Respiratory Viruses (2013). Available at: https://www.google.com/url?sa=t&rct=j&q=&esrc=s&source=web&cd=&ved=2ahUKEwiX29HW056BAxWCQzABHbagAwgQFnoECBYQAQ&url=https%3A%2F%2Fwww.paho.org%2Fes%2Ffile%2F36171%2Fdownload%3Ftoken%3DZxaiKn_7&usg=AOvVaw3UWDOuq8btw0ru5VT7XlyQ&opi=89978449

[17] Wilson CB Kollmann TR. Induction of antigen-specific immunity in human neonates and infants. Nestle Nutr Workshop Ser Pediatr Program. (2008) 61:183–95. 10.1159/000113493 18196952

[18] Corbett NP Blimkie D Ho KC Cai B Sutherland DP Kallos A Ontogeny of toll-like receptor mediated cytokine responses of human blood mononuclear cells. PLoS One. (2010) 5(11):e15041. 10.1371/journal.pone.0015041 21152080 PMC2994830

[19] PrabhuDas M Adkins B Gans H King C Levy O Ramilo O Challenges in infant immunity: implications for responses to infection and vaccines. Nat Immunol. (2011) 12(3):189–94. 10.1038/ni0311-189 21321588

[20] Kollmann TR Crabtree J Rein-Weston A Blimkie D Thommai F Wang XY Neonatal innate Tlr-mediated responses are distinct from those of adults. J Immunol. (2009) 183(11):7150–60. 10.4049/jimmunol.0901481 19917677 PMC4556237

[21] Halasa NB O’Shea A Shi JR LaFleur BJ Edwards KM. Poor immune responses to a birth dose of diphtheria, tetanus, and acellular pertussis vaccine. J Pediatr. (2008) 153(3):327–32. 10.1016/j.jpeds.2008.03.011 18534242 PMC3773719

[22] Knuf M Schmitt HJ Jacquet JM Collard A Kieninger D Meyer CU Booster vaccination after neonatal priming with acellular pertussis vaccine. J Pediatr. (2010) 156(4):675–8. 10.1016/j.jpeds.2009.12.019 20303444

[23] Cuenca AG Wynn JL Moldawer LL Levy O. Role of innate immunity in neonatal infection. Am J Perinatol. (2013) 30(2):105–12. 10.1055/s-0032-1333412 23297181 PMC3959733

[24] Dowling DJ Levy O. Ontogeny of early life immunity. Trends Immunol. (2014) 35(7):299–310. 10.1016/j.it.2014.04.007 24880460 PMC4109609

[25] Kollmann TR Levy O Hanekom W. Vaccine-induced immunity in early life. Vaccine. (2013) 31(21):2481–2. 10.1016/j.vaccine.2013.04.025 23588085 PMC4922257

[26] Pantell RH Roberts KB Adams WG Dreyer BP Kuppermann N O'Leary ST Evaluation and management of well-appearing febrile infants 8 to 60 days old. Pediatrics. (2021) 148(2):e2021052228. 10.1542/peds.2021-052228 34281996

[27] Nakaya HI Wrammert J Lee EK Racioppi L Marie-Kunze S Haining WN Systems biology of vaccination for seasonal influenza in humans. Nat Immunol. (2011) 12(8):786–95. 10.1038/ni.2067 21743478 PMC3140559

[28] Querec T Bennouna S Alkan S Laouar Y Gorden K Flavell R Yellow fever Vaccine Yf-17d activates multiple dendritic cell subsets via Tlr2, 7, 8, and 9 to stimulate polyvalent immunity. J Exp Med. (2006) 203(2):413–24. 10.1084/jem.20051720 16461338 PMC2118210

[29] Obermoser G Presnell S Domico K Xu H Wang Y Anguiano E Systems scale interactive exploration reveals quantitative and qualitative differences in response to influenza and pneumococcal vaccines. Immunity. (2013) 38(4):831–44. 10.1016/j.immuni.2012.12.008 23601689 PMC3681204

[30] Furman D Davis MM. New approaches to understanding the immune response to vaccination and infection. Vaccine. (2015) 33(40):5271–81. 10.1016/j.vaccine.2015.06.117 26232539 PMC4581990

[31] Furman D Jojic V Sharma S Shen-Orr SS Angel CJ Onengut-Gumuscu S Cytomegalovirus infection enhances the immune response to influenza. Sci Transl Med. (2015) 7(281):281ra43. 10.1126/scitranslmed.aaa2293 25834109 PMC4505610

[32] de St G F Webster RG. Disquisitions of original antigenic sin. I. Evidence in man. J Exp Med. (1966) 124(3):331–45. 10.1084/jem.124.3.331 5922742 PMC2138235

[33] Roltgen K Nielsen SCA Silva O Younes SF Zaslavsky M Costales C Immune imprinting, breadth of variant recognition, and germinal center response in human Sars-Cov-2 infection and vaccination. Cell. (2022) 185(6):1025–40 e14. 10.1016/j.cell.2022.01.018 35148837 PMC8786601

[34] Rothman AL. Immunity to dengue virus: a tale of original antigenic sin and tropical cytokine storms. Nat Rev Immunol. (2011) 11(8):532–43. 10.1038/nri3014 21760609

[35] Mejias A Dimo B Suarez NM Garcia C Suarez-Arrabal MC Jartti T Whole blood gene expression profiles to assess pathogenesis and disease severity in infants with respiratory syncytial virus infection. PLoS Med. (2013) 10(11):e1001549. 10.1371/journal.pmed.1001549 24265599 PMC3825655

[36] Hall CB Weinberg GA Iwane MK Blumkin AK Edwards KM Staat MA The burden of respiratory syncytial virus infection in young children. N Engl J Med. (2009) 360(6):588–98. 10.1056/NEJMoa0804877 19196675 PMC4829966

[37] Belkaid Y Harrison OJ. Homeostatic immunity and the microbiota. Immunity. (2017) 46(4):562–76. 10.1016/j.immuni.2017.04.008 28423337 PMC5604871

[38] Chapman TJ Pham M Bajorski P Pichichero ME. Antibiotic use and vaccine antibody levels. Pediatrics. (2022) 149(5):e2021052061. 10.1542/peds.2021-052061 35474546 PMC9648114

[39] Ramilo O Mejias A. Antibiotics and immunizations: a complex interaction. Pediatrics. (2022) 149(5):e2021055610. 10.1542/peds.2021-055610 35474544

[40] UNICEF. The state of the world’s children 2023. for every child, vaccination (2023). Available at: https://www.unicef.org/reports/state-worlds-children-2023

[41] Organización Mundial de la Salud. Maternal mortality (2023). Available at: https://www.who.int/news-room/fact-sheets/detail/maternal-mortality

[42] Castro A. Maternal and child mortality worsens in Latin America and the Caribbean. Lancet. (2020) 396(10262):e85. 10.1016/S0140-6736(20)32142-5 33065031 PMC7553735

[43] FAO, FIDA, OPS, WFP, UNICEF. América Latina Y el caribe—panorama regional De la seguridad Alimentaria Y nutricional 2021: EstadíSticas Y tendencias. United States: FAO, FIDA, OPS, WFP y UNICEF (2021). Available at: https://www.fao.org/3/cb7497es/cb7497es.pdf

[44] Organización Panamerica de la Salud. Nuevo Informe De La Onu: El Hambre En América Latina Y El Caribe Aumentó En 13,8 Millones De Personas En Solo Un Año (2021). Available at: https://www.paho.org/es/noticias/30-11-2021-nuevo-informe-onu-hambre-america-latina-caribe-aumento-138-millones-personas

[45] Meira CAR Buccini G Azeredo CM Conde WL Rinaldi AEM. Evolution of breastfeeding indicators and early introduction of foods in Latin American and Caribbean countries in the decades of 1990, 2000 and 2010. Int Breastfeed J. (2022) 17(1):32. 10.1186/s13006-022-00477-6 35459227 PMC9034574

[46] UNICEF. Unicef-who-the world bank: joint child malnutrition estimates — levels and trends—2020 edition (2020). Available at: https://data.unicef.org/resources/jme-report-2020/

[47] Serie Derechos Humanos Y Salud. Medio ambiente Y cambio climático: un enfoque basado en los derechos humanos. United States: Organización Panamerica de la Salud (2022). Available at: https://iris.paho.org/bitstream/handle/10665.2/55293/OPSLEGDHdhs7210001_spa.pdf?sequence=4&isAllowed=y

[48] Heng YY Asad I Coleman B Menard L Benki-Nugent S Hussein Were F Heavy metals and neurodevelopment of children in low and middle-income countries: a systematic review. PLoS One. (2022) 17(3):e0265536. 10.1371/journal.pone.0265536 35358213 PMC8970501

[49] Olivero-Verbel JA-O N Alcala-Orozco M Caballero-Gallardo K. Population exposure to lead and mercury in Latin America. Curr Opinion Toxicol. (2021) 27(5):27–37. 10.1016/j.cotox.2021.06.002

[50] Olympio KP Goncalves CG Salles FJ Ferreira AP Soares AS Buzalaf MA What are the blood lead levels of children living in Latin America and the Caribbean? Environ Int. (2017) 101:46–58. 10.1016/j.envint.2016.12.022 28159393

[51] Wright C Blake N Glennie L Smith V Bender R Kyu H The global burden of meningitis in children: challenges with interpreting global health estimates. Microorganisms. (2021) 9(2):377. 10.3390/microorganisms9020377 33668442 PMC7917636

[52] Le Govic Y Demey B Cassereau J Bahn YS Papon N. Pathogens infecting the central nervous system. PLoS Pathog. (2022) 18(2):e1010234. 10.1371/journal.ppat.1010234 35202445 PMC8870494

[53] Bottomley MJ Serruto D Safadi MA Klugman KP. Future challenges in the elimination of bacterial meningitis. Vaccine. (2012) 30(Suppl 2):B78–86. 10.1016/j.vaccine.2011.12.099 22607903

[54] Collaborators GBDDD. Estimates of global, regional, and national morbidity, mortality, and aetiologies of diarrhoeal diseases: a systematic analysis for the global burden of disease study 2015. Lancet Infect Dis. (2017) 17(9):909–48. 10.1016/S1473-3099(17)30276-1 28579426 PMC5589208

[55] Lopez-Medina E Parra B Davalos DM Lopez P Villamarin E Pelaez M. Acute gastroenteritis in a pediatric population from cali, Colombia in the post rotavirus vaccine era. Int J Infect Dis. (2018) 73:52–9. 10.1016/j.ijid.2018.06.006 29908961

[56] Foster YY Correoso Guevara JD Nuñez-Ortega JM. Factores de riesgo de enfermedad diarreica aguda en menores de 5 años. Revista Médico Científica. (2021) 34(1):1–8. 10.37416/rmc.v34i1.573

[57] Gentile A Bardach A Ciapponi A Garcia-Marti S Aruj P Glujovsky D Epidemiology of community-acquired pneumonia in children of Latin America and the Caribbean: a systematic review and meta-analysis. Int J Infect Dis. (2012) 16(1):e5–15. 10.1016/j.ijid.2011.09.013 22056731

[58] Comite de N, Comite de I, Comite de Medicina Interna P, Comite de Pediatria Ambulatoria y C, Comite de Pediatria A. Recommendations for the management of acute lower respiratory infections in children under 2 years of age. Update 2021. Arch Argent Pediatr. (2021) 119(4):S171–S97. 10.5546/aap.2021.S171 34309327

[59] UNHCR. Global trends report 2021: the un refugee agency (2021). Available at: https://www.unhcr.org/media/global-trends-report-2021

[60] Council On Environmental H. Global climate change and children’s health. Pediatrics. (2015) 136(5):992–7. 10.1542/peds.2015-3232 26504130

[61] United Nations Development Programme. Sustainable development goals—Sdgs and the 2030 Agenda (2023). Available at: https://www.undp.org/sustainable-development-goals?utm_source=EN&utm_medium=GSR&utm_content=US_UNDP_PaidSearch_Brand_English&utm_campaign=CENTRAL&c_src=CENTRAL&c_src2=GSR&gclid=Cj0KCQjwwtWgBhDhARIsAEMcxeDLCCG0Q5jp81irPm0ZxvAchGd-97OKdggu9jNQ3zep3IhJO76FsKAaAipwEALw_wcB

[62] Pan American Health Organization. Iniciativa Regional Para La Eliminación De La Transmisión Maternoinfantil Del Vih Y De La Sífilis Congénita En América Latina Y El Caribe: Documento Conceptual (2009). Available at: https://iris.paho.org/handle/10665.2/49406

[63] . . Acog committee opinion No. 762: prepregnancy counseling. Obstet Gynecol. (2019) 133(1):e78–89. 10.1097/AOG.0000000000003013 30575679

[64] FAO; IFAD; UNICEF; PAHO; WFP. Regional overview of food security and nutrition in latin america and the caribbean: towards improving affordability of healthy diets (2023). Available at: https://www.fao.org/documents/card/en/c/cc3859en

[65] Orjuela-Grimm M Deschak C Aragon Gama CA Bhatt Carreno S Hoyos L Mundo V Migrants on the move and food (in) security: a call for research. J Immigr Minor Health. (2022) 24(5):1318–27. 10.1007/s10903-021-01276-7 34542776 PMC8450693

[66] Centers for Disease C. Rubella vaccination during pregnancy—United States, 1971–1988. MMWR Morb Mortal Wkly Rep. (1989) 38(17):289–93. 2496289

[67] Hamkar R Jalilvand S Abdolbaghi MH Esteghamati AR Hagh-Goo A Jelyani KN Inadvertent Rubella vaccination of pregnant women: evaluation of possible transplacental infection with Rubella vaccine. Vaccine. (2006) 24(17):3558–63. 10.1016/j.vaccine.2006.02.001 16510217

[68] Bar-Oz B Levichek Z Moretti ME Mah C Andreou S Koren G. Pregnancy outcome following Rubella vaccination: a prospective controlled study. Am J Med Genet A. (2004) 130A(1):52–4. 10.1002/ajmg.a.30225 15368496

[69] Center for Disease Control and Prevention. Covid-19 vaccines while pregnant or breastfeeding (2022). Available at: https://www.cdc.gov/coronavirus/2019-ncov/vaccines/recommendations/pregnancy.html#:∼:text=CDC%20recommends%20COVID%2D19%20vaccines,become%20pregnant%20in%20the%20future

[70] Sudarshan MK Giri MS Mahendra BJ Venkatesh GM Sanjay TV Narayana DH Assessing the safety of post-exposure rabies immunization in pregnancy. Hum Vaccin. (2007) 3(3):87–9. 10.4161/hv.3.3.4010 17375003

[71] Manning SE Rupprecht CE Fishbein D Hanlon CA Lumlertdacha B Guerra M Human rabies prevention--United States, 2008: recommendations of the advisory committee on immunization practices. MMWR Recomm Rep. (2008) 57(RR-3):1–28. 18496505

[72] Cavalcanti DP Salomao MA Lopez-Camelo J Pessoto MA, Campinas Group of Yellow Fever Immunization during P. Early exposure to yellow fever vaccine during pregnancy. Trop Med Int Health. (2007) 12(7):833–7. 10.1111/j.1365-3156.2007.01851.x 17596249

[73] World Health Organization. Vaccines and vaccination against yellow fever. Who position paper—June 2013. Wkly Epidemiol Rec. (2013) 88(27):269–83. 23909008

[74] World Health Organization. Immunization coverage (2023). Available at: https://www.who.int/news-room/fact-sheets/detail/immunization-coverage

[75] Pan American Health Organization. Immunization in the Americas: 2022 summary (2022). Available at: https://www.paho.org/en/documents/immunization-americas-2022-summary

[76] Castrejon MM Leal I de Jesus Pereira Pinto T Guzman-Holst A. The impact of COVID-19 and catch-up strategies on routine childhood vaccine coverage trends in Latin America: a systematic literature review and database analysis. Hum Vaccin Immunother. (2022) 18(6):2102353. 10.1080/21645515.2022.2102353 36084255 PMC9746494

[77] Aguero ML A Castillo JBD Falleiros-Arlant LH Berezin E de Moraes JC Torres-Martinez C Risks of low vaccination coverage and strategies to prevent the resurgence of vaccine-preventable diseases in infants in the COVID-19 pandemic scenario: recommendations for Latin America and the Caribbean by the group of experts on infant immunization for Latin America. Expert Rev Vac. (2023) 22(1):1091–101. 10.1080/14760584.2023.2271057 37843489

[78] Sachs JD Karim SSA Aknin L Allen J Brosbol K Colombo F The lancet commission on lessons for the future from the COVID-19 pandemic. Lancet. (2022) 400(10359):1224–80. 10.1016/S0140-6736(22)01585-9 36115368 PMC9539542

[79] Castellanos LGG Kurtis H Mello MB Perez F Ropero AM Salvatella R Etmi Plus—Marco Para La Eliminación De La Transmisión Maternoinfantil Del Vih, La Sífilis, La Hepatitis Y La Enfermedad De Chagas (2017). Available at: www.paho.org/hq/dmdocuments/2017/2017-cha-etmi-plus-marco-vihhep-chagas.pdf

[80] Pan American Health Organization. Epidemiological review of Syphilis in the Americas (2021). Available at: https://www.paho.org/en/documents/epidemiological-review-syphilis-americas-december-2021

[81] Verani JR McGee L Schrag SJ. Division of bacterial diseases NCfI, respiratory diseases CfDC, prevention. prevention of perinatal group B streptococcal disease-revised guidelines from Cdc, 2010. MMWR Recomm Rep. (2010) 59(RR-10):1–36. 21088663

[82] Stillwaggon E Carrier CS Sautter M McLeod R. Maternal serologic screening to prevent congenital toxoplasmosis: a decision-analytic economic model. PLoS Negl Trop Dis. (2011) 5(9):e1333. 10.1371/journal.pntd.0001333 21980546 PMC3181241

[83] Lappalainen M Sintonen H Koskiniemi M Hedman K Hiilesmaa V Ammala P Cost-benefit analysis of screening for toxoplasmosis during pregnancy. Scand J Infect Dis. (1995) 27(3):265–72. 10.3109/00365549509019020 8539552

[84] Foulon W Naessens A Ho-Yen D. Prevention of congenital toxoplasmosis. J Perinat Med. (2000) 28(5):337–45. 10.1515/JPM.2000.043 11125923

[85] Group SS, Thiebaut R Leproust S Chene G Gilbert R. Effectiveness of prenatal treatment for congenital toxoplasmosis: a meta-analysis of individual patients’ data. Lancet. (2007) 369(9556):115–22. 10.1016/S0140-6736(07)60072-5 17223474

[86] Manicklal S Emery VC Lazzarotto T Boppana SB Gupta RK. The "silent" global burden of congenital cytomegalovirus. Clin Microbiol Rev. (2013) 26(1):86–102. 10.1128/CMR.00062-12 23297260 PMC3553672

[87] Morton CC Nance WE. Newborn hearing screening-a silent revolution. N Engl J Med. (2006) 354(20):2151–64. 10.1056/NEJMra050700 16707752

[88] Adler SP Marshall B. Cytomegalovirus infections. Pediatr Rev. (2007) 28(3):92–100. 10.1542/pir.28-3-92 17332168

[89] Orfanos I Elfving K Sotoca Fernandez J Wennlund L Weiber S Eklund EA Management and outcome of febrile infants </=60 days, with emphasis on infants </=21 days old, in Swedish pediatric emergency departments. Pediatr Infect Dis J. (2022) 41(7):537–43. 10.1097/INF.0000000000003542 35389959

[90] Byington CL Enriquez FR Hoff C Tuohy R Taggart EW Hillyard DR Serious bacterial infections in febrile infants 1 to 90 days old with and without viral infections. Pediatrics. (2004) 113(6):1662–6. 10.1542/peds.113.6.1662 15173488

[91] Furfaro LL Chang BJ Payne MS. Perinatal streptococcus agalactiae epidemiology and surveillance targets. Clin Microbiol Rev. (2018) 31(4):e00049–18. 10.1128/CMR.00049-18 30111577 PMC6148196

[92] Cruz-Hernandez MG-G Garcia Garcia JJ Cruz-Martinez O Mintegi-Raso S Moreno-Villares JM. Manual De pediatría. Spain: Ergon (2019). Available at: https://dialnet.unirioja.es/servlet/libro?codigo=831916

[93] Picciano MF. Human milk: nutritional aspects of a dynamic food. Biol Neonate. (1998) 74(2):84–93. 10.1159/000014015 9691151

[94] Field CJ. The immunological components of human milk and their effect on immune development in infants. J Nutr. (2005) 135(1):1–4. 10.1093/jn/135.1.1 15623823

[95] Legrand D. Overview of lactoferrin as a natural immune modulator. J Pediatr. (2016) 173:Suppl:S10-5. 10.1016/j.jpeds.2016.02.071 27234406

[96] Wada Y Lonnerdal B. Bioactive peptides derived from human milk proteins-mechanisms of action. J Nutr Biochem. (2014) 25(5):503–14. 10.1016/j.jnutbio.2013.10.012 24411973

[97] Ng TB Cheung RC Wong JH Wang Y Ip DT Wan DC Antiviral activities of whey proteins. Appl Microbiol Biotechnol. (2015) 99(17):6997–7008. 10.1007/s00253-015-6818-4 26198883 PMC7080083

[98] Drago-Serrano ME Campos-Rodriguez R Carrero JC de la Garza M. Lactoferrin: balancing ups and downs of inflammation due to microbial infections. Int J Mol Sci. (2017) 18(3):501. 10.3390/ijms18030501 28257033 PMC5372517

[99] Brandtzaeg P. The mucosal immune system and its integration with the mammary glands. J Pediatr. (2010) 156(2 Suppl):S8–15. 10.1016/j.jpeds.2009.11.014 20105666

[100] Fernandez L Langa S Martin V Maldonado A Jimenez E Martin R The human milk Microbiota: origin and potential roles in health and disease. Pharmacol Res. (2013) 69(1):1–10. 10.1016/j.phrs.2012.09.001 22974824

[101] Henkle E Steinhoff MC Omer SB Roy E Arifeen SE Raqib R The effect of exclusive breast-feeding on respiratory illness in young infants in a maternal immunization trial in Bangladesh. Pediatr Infect Dis J. (2013) 32(5):431–5. 10.1097/INF.0b013e318281e34f 23249922

[102] Schlaudecker EP Steinhoff MC Omer SB McNeal MM Roy E Arifeen SE Iga and neutralizing antibodies to influenza a virus in human milk: a randomized trial of antenatal influenza immunization. PLoS One. (2013) 8(8):e70867. 10.1371/journal.pone.0070867 23967126 PMC3743877

[103] Baird JK Jensen SM Urba WJ Fox BA Baird JR. Sars-Cov-2 antibodies detected in mother’s milk post-vaccination. J Hum Lact. (2021) 37(3):492–8. 10.1177/08903344211030168 34297643 PMC8685565

[104] Jones G Steketee RW Black RE Bhutta ZA Morris SS. Bellagio child survival study G. How many child deaths can we prevent this year? Lancet. (2003) 362(9377):65–71. 10.1016/S0140-6736(03)13811-1 12853204

[105] Horta BLV Victora CG. Long-Term effects of breastfeeding: a systematic review. United States: World Health Organization (2013). Available at: https://apps.who.int/iris/bitstream/handle/10665/79198/9789241505307_eng.pdf;jsessionid=04B821DB34FBCA65AC93879FF0AA5978?sequence=1

[106] Centers for Disease Control and Prevention. Antibiotic prescribing and use (2023). Available at: https://www.cdc.gov/antibiotic-use/index.html

